# Biomineralization of calcium carbonates and their engineered applications: a review

**DOI:** 10.3389/fmicb.2013.00314

**Published:** 2013-10-29

**Authors:** Navdeep K. Dhami, M. Sudhakara Reddy, Abhijit Mukherjee

**Affiliations:** ^1^Department of Biotechnology, Thapar UniversityPatiala, India; ^2^Department of Civil Engineering, Indian Institute of TechnologyGandhinagar, India

**Keywords:** biomineralization, bacteria, urease, biofilm, extrapolymeric substances, calcite

## Abstract

Microbially induced calcium carbonate precipitation (MICCP) is a naturally occurring biological process in which microbes produce inorganic materials as part of their basic metabolic activities. This technology has been widely explored and promising with potential in various technical applications. In the present review, the detailed mechanism of production of calcium carbonate biominerals by ureolytic bacteria has been discussed along with role of bacteria and the sectors where these biominerals are being used. The applications of bacterially produced carbonate biominerals for improving the durability of buildings, remediation of environment (water and soil), sequestration of atmospheric CO_2_ filler material in rubbers and plastics etc. are discussed. The study also sheds light on benefits of bacterial biominerals over traditional agents and also the issues that lie in the path of successful commercialization of the technology of microbially induced calcium carbonate precipitation from lab to field scale.

## Introduction

**Figure 1:**
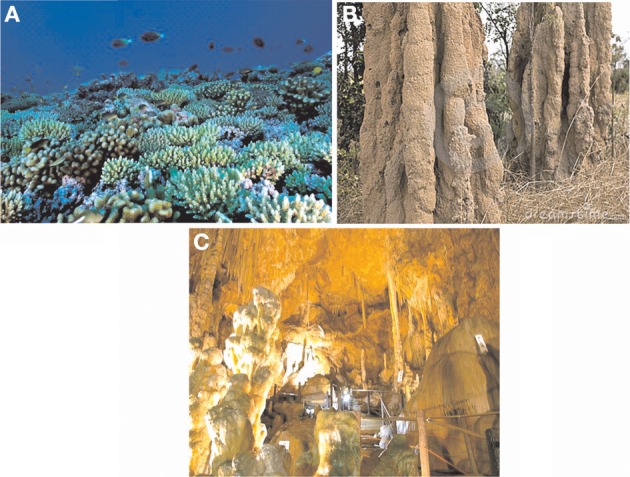
**Bio-mineralization of calcium carbonates in natural structures (A) Corals (B) Ant hills (C) Limestone caves (www.sciencedaily.com, www.indiamike.com, www.wikipedia.org)**.

“Biominerals are everywhere.” If we take a look around, we see ourselves surrounded by biominerals whether in the form of beautiful corals, ant hills, caves, shells of mollusks, teeth, bones or rocks (Figure 1). Researchers around the globe are now focusing on harnessing the technical applications of these biominerals in various fields.

Biomineralization is a process by which living organisms produce minerals. These could be silicates in algae and diatoms, carbonates in invertebrates and calcium, phosphates and carbonates in vertebrates. The synthesis of minerals by prokaryotes is broadly classified into two classes: Biologically controlled mineralization (BCM) and Biologically induced mineralization (BIM) (Lowenstam, 1981; Lowenstam and Weiner, 1989). Minerals are directly synthesized at a specific location within or on the cell and only under certain conditions in case of BCM but in case of biologically induced mineralization, the minerals are formed extracellularly as a result of metabolic activity of the organism. The extracellular production of these biominerals invited scientists worldwide for harnessing this capability of microbes for various bioengineering applications.

Minerals known to be formed via biologically induced mineralization through passive surface-mediated mineralization include Fe, Mn, and other metal oxides, e.g., ferrihydrite (5Fe_2_O_3_·9H_2_O), hematite (α-Fe_2_O_3_), and goethite (α-FeOOH); metal sulfates, phosphates, and carbonates; phosphorite; Fe and Fe-Al silicates; and metal sulfides. Of all the minerals that have been associated with biomineralization, carbonates are the most obvious. Microbially induced calcium carbonate precipitation (MICCP), most widely studied branch of biomineralization holds promise for variety of fields ranging from Biotechnology, Geotechnology, Paleobiology to Civil Engineering. It has implications for: (1) atmospheric CO_2_ fixation through carbonate sediment formation and lithification (Krumbein, 1979; Monger et al., 1991; Chafetz and Buczynski, 1992; Folk, 1993) and dolomite precipitation (Vasconcelos et al., 1995) (2) solid-phase capture of inorganic contaminants (Warren et al., 2001) (3) pathological formation of mineral concretions, such as gallstones and kidney stones in humans (Keefe, 1976) (4) the possibility of understanding extraterrestrial biological processes such as Martian carbonate production by bacteria (McKay et al., 1996; Thomas-Keprta et al., 1998).

### Microbially induced CaCO_3_ precipitation

Calcium carbonate precipitation is a rather straightforward chemical process governed mainly by four key factors: (1) the calcium concentration, (2) the concentration of dissolved inorganic carbon (DIC), (3) the pH and (4) the availability of nucleation sites (Hammes and Verstraete, 2002). CaCO_3_ precipitation requires sufficient calcium and carbonate ions so that the ion activity product (IAP) exceeds the solubility constant (*K*_so_) [Equations (1) and (2)]. From the comparison of the IAP with the *K*_so_, the saturation state (Ω) of the system can be defined: if Ω > 1 the system is oversaturated and precipitation is likely (Morse, 1983) as:

(1) $${\text{Ca}}^{2+}+{\text{CO}}_{3}^{2-}↔{\text{CaCO}}_{3}$$

(2) $$\Omega =a({\text{Ca}}^{2+})a({\text{CO}}_{3}^{2-})/K_{\text{so}}\text{ with }K_{\text{so calcite},\;{\text{25}}^{\text{o}}}=4.8\times 10^{-9}\;\;$$

The concentration of carbonate ions is related to the concentration of DIC and the pH of a given aquatic system. In addition, the concentration of DIC depends on several environmental parameters such as temperature and the partial pressure of carbon dioxide (for systems exposed to the atmosphere). The equilibrium reactions and constants governing the dissolution of CO_2_ in aqueous media (25°C and 1 atm) are given in Equations (3)–(6) (Stumm and Morgan, 1981):

(3) $${\text{CO}}_{2(g)}↔{\text{CO}}_{2(\text{aq}.)}(pK_{H}=1.468)$$

(4) $${\text{CO}}_{2(\text{aq}.)}+{\text{H}}_{2}\text{O}↔{\text{H}}_{2}{\text{CO}}_{3}*(pK=2.84)$$

(5) $${\text{H}}_{2}{\text{CO}}_{3}*↔{\text{H}}^{+}+{\text{HCO}}_{3}^{-}(pK1=6.352)$$

(6) $${\text{HCO}}_{3}^{-}↔{\text{CO}}_{3}^{2-}+{\text{H}}^{+}(pK2=10.329)$$

With H_2_CO_3^*^_ = CO_2(aq.)_ + H_2_CO_3_.

Under natural conditions, the precipitation of carbonates occurs very slowly over long geological times but in order to produce large amounts of carbonates shortly there is need to look for microbes with the ability to create conditions for precipitation of carbonates in shorter times. Different bacterial species precipitate carbonates in alkaline environments rich in Ca^2+^ ions and various mechanisms which could induce precipitation by bacteria in natural habitats have been proposed (Ehrlich, 1996; Rivadeneyra et al., 2004). Though, the precise role of bacteria and bacterial activities in carbonate crystallization remains unclear, they seem to fall in 3 categories:

- As per the first hypothesis, mineralization occurs as a byproduct of microbial metabolism (Rivadeneyra et al., 1994; Douglas and Beveridge, 1998; Castanier et al., 1999; Lian et al., 2006);
- As per the second hypothesis, carbonate nucleation takes place on the cell wall (Rivadeneyra et al., 1998; Castanier et al., 2000) and
- Lastly, the third hypothesis involves role of extracellular macromolecules (Ercole et al., 2007; Decho, 2009).

Mainly four groups of microorganisms are seen to be involved in the process

(i) Photosynthetic organisms—such as cyanobacteria and algae
(ii) Sulphate reducing bacteria—that are responsible for dissimilatory reduction of sulphates
(iii) Organisms utilizing organic acids
(iv) Organisms that are involved in the nitrogen cycle either ammonification of amino acids/ nitrate reduction/ hydrolysis of urea (Stocks-Fischer et al., 1999; Hammes and Verstraete, 2002).

Of all the above, Microbially induced calcium carbonate precipitation (MICCP) via urea hydrolysis is the simplest and most widely used method for precipitation of carbonates for several technical applications.

### MICCP via urea hydrolysis

The precipitation of carbonates via urea hydrolysis by ureolytic bacteria is the most straightforward and most easily controlled mechanism of MICCP with potential to produce high amounts of carbonates in short period of time. During microbial urease activity, 1 mol of urea is hydrolyzed intracellularly to 1 mol of ammonia and 1 mol of carbonate (Equation 7), which spontaneously hydrolyzes to form additional 1 mol of ammonia and carbonic acid (Equation 8) as follows:

(7) $$\text{CO}{\left({\text{NH}}_{2}\right)}_{2}{\text{+H}}_{2}\text{O}\underset{\to }{\text{bacteria}}{\text{NH}}_{2}{\text{COOH+NH}}_{3}$$

(8) $${\text{NH}}_{2}{\text{COOH+H}}_{2}\text{O}\to {\text{NH}}_{3}{\text{+H}}_{2}{\text{CO}}_{3}$$

These products equilibrate in water to form bicarbonate, 1 mol of ammonium and hydroxide ions which give rise to pH increase

(9) $${\text{H}}_{2}{\text{CO}}_{3}\to {\text{2H}}^{+}{\text{+2CO}}_{3}^{2-}$$

(10) $${\text{NH}}_{3}{\text{+H}}_{2}\text{O}\to {\text{NH}}^{4+}{\text{+OH}}^{-}$$

(11) $${\text{Ca}}^{2+}{\text{+CO}}_{3}^{2-}\to {\text{CaCO}}_{3}(K_{SP}=3.8\times 10^{-9})$$

*K*_*SP*_ is the solubility product in Equation 11.

**Figure 2:**
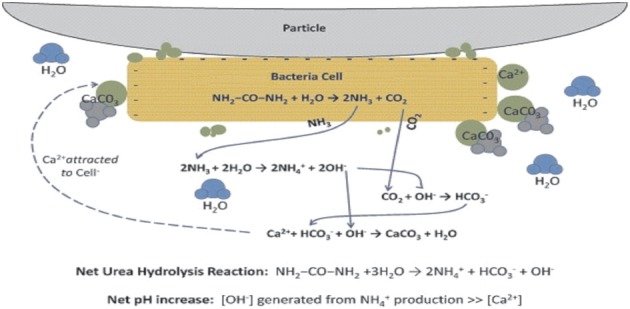
**Bacteria serving as nucleation site for CaCO_3_ precipitation in the sand particles (Source: DeJong et al., 2010)**.

The primary role of bacteria has been ascribed to their ability to create an alkaline environment through various physiological activities (Figure 2). Bacterial surfaces also play an important role in calcium precipitation (Fortin et al., 1997). Due to the presence of several negatively charged groups, at a neutral pH, positively charged metal ions could be bound on bacterial surfaces, favoring heterogenous nucleation (Douglas and Beveridge, 1998; Bäuerlein, 2003). Commonly, carbonate precipitates develop on the external surface of bacterial cells by successive stratification (Pentecost and Bauld, 1988; Castanier et al., 1999) and bacteria can be embedded in growing carbonate crystals (Rivadeneyra et al., 1998; Castanier et al., 1999).

Possible biochemical reactions in urea-CaCl_2_ medium to precipitate CaCO_3_ at the cell surface can be summarized as follows:

(12) $${\text{Ca}}^{2+}+\text{Cell}\to {\text{Cell-Ca}}^{2+}$$

(13) $${\text{Cl}}^{-}{\text{+HCO}}^{3-}+{\text{NH}}_{3}\to {\text{NH}}_{4}{\text{Cl+CO}}_{3}^{2-}$$

(14) $$\text{Cell}-{\text{Ca}}^{2+}+{\text{CO}}_{3}^{2-}\to \text{Cell}-{\text{CaCO}}_{3}$$

### Enzyme urease

Several authors have investigated various conditions for calcium carbonate precipitation via urea hydrolysis (Table 1). The actual role of the bacteria in the precipitation remains, however, a matter of debate. Some authors believe this precipitation to be an unwanted and accidental by-product of the metabolism (Knorre and Krumbein, 2000) while others think that it is a specific process with ecological benefits for the precipitating organisms (Ehrlich, 1996; Mc Connaughey and Whelan, 1997). These bacteria include *Bacillus pasteurii*, *Pseudomonas* sp., *Variovorax* sp., *Leuconostoc mesenteroides, Micrococcus* sp., *Bacillus subtilis*, *Deleya halophila*, *Halomonas eurihalina* and *Myxococcus xanthus* (Mobley and Hausinger, 1989; Rivadeneyra et al., 1991, 1996, 1998; Ferris and Stehmeier, 1992; Stocks-Fischer et al., 1999; Tiano et al., 1999; Castanier et al., 2000; Fujita et al., 2000; Rodriguez-Navarro et al., 2003).

**Table 1:** **Reaction conditions reported in the literature for production of CaCO_3_ via urea hydrolysis**.

**Purpose**	**Urea (mM)**	**Ca^2+^ (mM)**	**Urease activity (mM/min)**	**Reference**
Sr^90^ sequestration	333	25	0.045	Fujita et al., 2000
Sr^90^ sequestration	330	0.025	0.042	Warren et al., 2001
Removal of Ca^2+^ from waste water	16	14	0.293	Hammes et al., 2003
Removal of Ca^2+^ from waste water	8	15	0.032	Hammes, 2002
Stone remediation	333	12–50	0.110	Stocks-Fischer et al., 1999
	333	340	0.02–0.12	De Muynck et al., 2011
Stone remediation	66	25	0.041	Bachmeier et al., 2002
Portland cement remediation	333	50	n/s	Ramachandran, 2001
Plugging of rock pores	333	0.25	n/s	Gollapudi et al., 1995
Biocementation	1500	1500	4–18	Whiffin, 2004
Sand column cementation	333	25	0.65	Achal et al., 2009a; Van Paassen, 2009
Soil cementation	1000	1000	n/s	Cheng et al., 2013
Biodeposition	333	340	n/s	De Belie and De Muynck, 2008
Carbonate precipitation	666	250	n/s	Okwadha and Li, 2010

### Polymorphism of carbonate crystals

**Figure 3:**
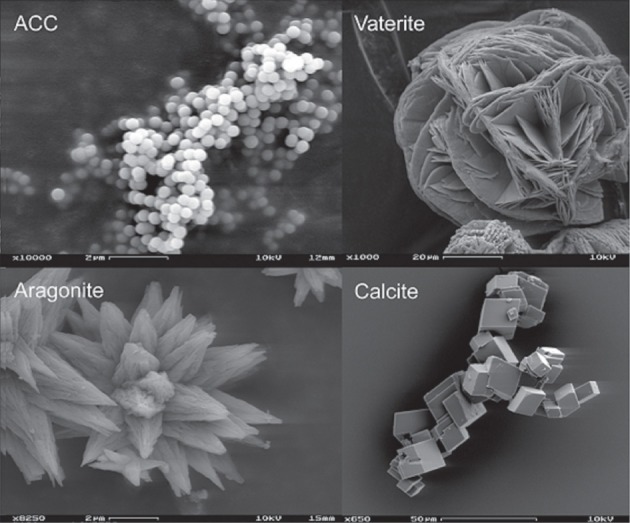
**Polymorphs of CaCO_3_ (http://www.ruhr-uni-bochum.de/sediment/forschung.html)**.

Studies have reported that bacterial induced calcium carbonate precipitation results in the production of different phases of CaCO_3_ (Rodriguez-Navarro et al., 2012; Rusznyak et al., 2012; Dhami et al., 2013a). Calcium carbonate forms three anhydrous polymorphs: calcite, aragonite and vaterite, two hydrated crystalline phases: monohydrocalcite (CaCO_3_·H_2_O) and ikaite (CaCO_3_·6H_2_O), and various amorphous phases (ACC) with differences in short range order and degree of hydration (Somasundaran and Agar, 1967; Lippmann, 1973; Rieger et al., 2007; Gower, 2008; Gebauer et al., 2010) (Figure 3). Although vaterite and calcite are the most common bacterial calcium carbonate polymorphs (Ben Chekroun et al., 2004; Rodriguez-Navarro et al., 2007; González-Muñoz et al., 2011), mineralization of monohydrocalcite (Krumbein, 1979) and aragonite (Krumbein, 1974; Sánchez-Navas et al., 2009) have also been reported. The evidence that bacterial mineralization of calcium carbonate involves the formation of ACC precursor phases is also growing (Hammes et al., 2003; Benzerara et al., 2006; Chen et al., 2009). The species specific precipitation of carbonate biominerals by various bacterial isolates has also been reported by many (Hammes et al., 2003; Rusznyak et al., 2012; Dhami et al., 2013a). But despite extensive studies on bacterial carbonatogenesis, little is known on what is the cause(s) of polymorph selection during bacterial calcium carbonate mineralization. Studies suggested that phase, amount and morphology of calcium carbonate minerals depend on supersaturation, temperature, pH and [Ca^2+^] / [CO^−^_32_] ratio. The saturation index, *SI* = log Ω = log IAP/*K*_*s*_; where Ω is the saturation state of the system, IAP is the ion activity product and K_*s*_ is the thermodynamic solubility product of the relevant phase. Calcium carbonate precipitation in microbial systems typically occurs when the saturation index (with respect to calcite) is above 1 (Arp et al., 2001; Mitchell and Ferris, 2006). Along with this, organics also play important role in the carbonate precipitation. The organics act as crystallization inhibitors (when in solution) and prevent the nucleation of calcium carbonate even at high SI values (Rodriguez-Navarro et al., 2007). It follows that bacterial presence and activity are a prerequisite for the precipitation of calcium carbonate. The bacterial metabolic activity produces the necessary increase in supersaturation as to induce the heterogeneous crystallization of calcium carbonate.

Besides its scientific interest, calcium carbonate polymorph selection can have important technical implications (Meldrum and Cölfen, 2008). For instance, the bacterial conservation of building materials requires the formation of a coherent, durable, calcium carbonate cement i.e., calcite. This is not fully achieved if metastable vaterite (more soluble than calcite) is formed during a bacterial conservation treatment (Rodriguez-Navarro et al., 2003).

## Applications of biominerals

The use of bacterially induced carbonate biominerals is becoming increasingly popular day by day. From removal of heavy metals and radionucleotides, removal of calcium from wastewater and biodegradation of pollutants, atmospheric CO_2_ sequestration, remediation of building materials, modifying the properties of soil and filler in rubber and plastics to fluorescent markers in stationery ink, bacterial carbonates are serving many fields.

### Removal of heavy metals and radionuclide

Heavy metals and radionuclides pose greatest challenges for their disposal. They are threatening not only to human health but to the entire environment. Remediation of ground water from radionuclides has been the topic of immediate attention and several measures have being taken for it (Warren et al., 2001; Fujita et al., 2004, 2008, 2010; Wu et al., 2011). Traditional methods of using pump and treat have been found ineffective (Fujita et al., 2008) and paved way for applications of ureolytic bacteria capable of CaCO_3_ precipitation. In this, biomineralization of radionuclide and contaminant metals into calcite occurs as competitive co-precipitation reaction in which suitable divalent cations are incorporated into the calcite lattice.

(15) $${}^{90}{\text{Sr}}^{2+}+{\text{OH}}^{-}+{\text{HCO}}_{3}^{-}={\text{SrCO}}_{3}\left(\text{s}\right)+{\text{H}}_{2}\text{O}$$

These cations and radionuclides integrate into the calcite structure via substitution of calcium ions in the microenvironment of the mineral precipitate, forming low - strontium carbonate minerals which have very low solubility (Fujita et al., 2000, 2004; Smith et al., 2004; Colwell et al., 2005; Mitchell and Ferris, 2006). Warren et al. (2001) reported 95% capturing of the total strontium in solid phase by Microbially induced calcite precipitation (MICP) with application of *S. pasteurii*. Fujita et al. (2008) successfully investigated the potential of enriching native ureolytic organism *in situ* for remediation of ground water by co-precipitation of Strontium.

Metal contamination of soil is especially problematic because of strong adsorption of many metals to the surfaces of soil particles. For removal of toxic metals, the conventional methodologies include phytoremediation, on site chemical leaching of contaminants, bioremediation by toxic metal tolerant bacterial species (Achal et al., 2012) but all these treatments are expensive and not successful for long terms. Other treatments include addition of cement or chemical fixatives, by capping with asphalt, or by *in situ* vitrification and excavation and confinement of soils in hazardous waste facilities (Schleck, 1990). Although rapid in effect, both of these options are expensive ($30–300/m^3^) and destroy the soil's future productivity (Cunningham et al., 1995).

MICCP has come up as a novel solution to this problem. Recently Achal et al. (2012) reported the positive potential of *Sporosarcina ginsengisoli* CR 5 for remediation of Arsenic contaminated sites as well as copper bioremediation by *Kocuria flava* CR1 (Achal et al., 2011b). Kurmaç (2009) also investigated the impact of varying concentrations of different metals as lead, cadmium, chromium, zinc, copper and nickel in MICP technology and reported that metal toxicity do play important role in microbial substrate degradation.

### Removal of calcium ions and polychlorinated biphenyls (PCBs)

The presence of a high concentration of calcium ions (500–1500 mgL^−1^) in the wastewater causes severe scaling in the pipelines and reactors due to calcium formation as carbonate, phosphate and/or gypsum. Hammes et al. (2003) studied the potential of removing Ca^2+^ ions from industrial wastewaters by MICCP. By the addition of a low concentration of urea (0–16 gL^−1^), up to 90% of the calcium ions was successfully removed from the examined wastewater.

The last 15 years have seen an increase in the types of contaminants to which bioremediation is being applied, including polychlorinated biphenyls (PCBs). Microbial processes are beginning to be used in the cleanup of these recalcitrant contaminants. The conventional methods of removing PCBs include solvent washing, hydroblasting, or sandblasting followed by encapsulation in epoxy coating. But all these treatments are quite inconvenient and ineffective due to resurfacing of the oil over time. An alternative to these is the use of carbonate biomineralizing bacterial isolates. Okwadha and Li (2010) recently reported the positive potential of ureolytic *S. pasteurii* cultures and urea/calcium for treatment of PCB - coated cement cylinders leading to surficial encapsulation of PCB containing oils.

### Carbon dioxide sequestration

The exponentially growing concentrations of carbon dioxide in the environment invited researchers from worldwide to deal with the problem. Different ways were proposed to reduce the emission of CO_2_ into the atmosphere and earlier strategies developed were:

- Reduce its production and release into the atmosphere (by use of less carbon intensive energy sources like wind, solar and nuclear energy)
- Increase efficiency of energy use from production to its end use.

But both these options were not possible practically, so researchers shifted to capturing/ sequestering the CO_2_ in a safe manner. Conversion of CO_2_ to carbonates offers the possibility of safe, stable and environmentally benign product for long term carbon sequestration, as massive carbonate mineral reservoirs have existed for millions of years. This option was looked upon by various researchers to capture and dispose the produced CO_2_ in a safe manner i.e., sequestration of CO_2_ (Herzog and Drake, 1996; Reichle et al., 1999; Shaffer, 2010; Sharma and Bhattacharya, 2010). This method includes fixing the CO_2_ in the form of carbonate minerals such as calcite, magnesite, and dolomite and came out to be safe and permanent method for disposing off the CO_2_ as the carbonate minerals are environmentally benign and stable. The proposed mechanism for reducing emissions is the capture and storage of CO_2_ in deep geologic reservoirs, such as deep saline aquifers. Leakage of CO_2_ back has also to be prevented which might be due to decreased well bore integrity and increased cap rock permeability (Barlet-Gouédard et al., 2009; Carey et al., 2010).

In nature, the sequestration of CO_2_ is based on the chemical fixation of CO_2_ in the form of carbonate minerals such as calcite, aragonite, dolomite and magnesite over a geological time scale. Scientists therefore proposed biomimetic CO_2_ sequestration by using biological catalysts like carbonic anhydrase (CA) to minimize the localized CO_2_ concentration. CA enzyme is ubiquitously distributed in organisms and fundamental to many eukaryotic biological processes like photosynthesis, respiration, CO_2_ and ion transport etc. (Smith and Ferry, 2000). Liu et al. (2005) used bovine CA as enzyme to accelerate CO_2_ hydration and reported that precipitation of calcium carbonate occurred much more quickly in presence of bovine CA. The role of biological CA in biological calcification of marine invertebrates (mollusks), fish otoliths, corals, hard tissues of vertebrates has been widely studied but applications of these microbes for CO_2_ sequestrations have started only recently (Beier and Anken, 2006; Tambutté et al., 2007).

MICCP serves as potential solution to seal fractures and high permeability leaking zones. The storage of CO_2_ is enhanced by MICCP by increasing the dissolved CO_2_ (as carbonate or bicarbonate) in the subsurface formation water or the precipitation of dissolved CO_2_ in carbonate minerals (Mitchell et al., 2010). Sequestration of CO_2_ by bacterial CA from different bacterial isolates has been recently shown by many (Ramanan et al., 2009; Wanjari et al., 2011; Yadav et al., 2011). Mitchell et al. (2010) and Phillips et al. (2013) proposed MICP to protect the well cements from supercritical CO_2_, plug microfractures near well environment and reduce the permeability in cap rock. Dupraz et al. (2009a,b) also investigated *S. pasteurii* in artificial groundwater to determine the transformation of CO_2_ into a solid carbonate phase (mineral trapping). Mitchell et al. (2010) demonstrated that as pH increases, the DIC increases and CO_2_ (g) decreases. It was concluded that ureolysis-driven MICCP in the subsurface can potentially increase the security of long-term CO_2_ storage. Kupriyanova et al. (2007) studied successful deposition of CaCO_3_ by extracellular CAs of *M. chthonoplastes* cyanobacterial cells. Recently Jansson and Northen (2010) reported potential employment of cyanobacteria for point-source carbon capture and sequestration. The cyanobacteria utilize solar energy through photosynthesis to convert carbon dioxide to recalcitrant calcium carbonate biominerals (Kamennaya et al., 2012).

### Filler for rubber, plastics and ink

Application of MICCP technique has been recently reported to produce a material that can be used as filler in rubber and plastics, fluorescent particles in stationery ink, and a fluorescent marker (Yoshida et al., 2010). The group isolated moderately thermophilic bacterium *Geobacillus thermoglucosidasius* from thermophilically composted organic waste. This bacterium catalyzes the formation of calcite crystals which harbor the property of fluorescence. The calcite crystals formed by *G. thermoglucosidasius* nucleation are excited by a wavelength interval of 260–400 nm, and their emission wavelengths are from 350 to 600 nm. The wide emission wavelength interval is a novel fluorescence property of *G. thermoglucosidasius* catalyzed calcite crystals which encourages it usage as filler in rubber and plastics, fluorescent particles in stationery ink, and fluorescent marker. In materials engineering, environmentally friendly systems with minimal energy consumption and resource depletion are required for producing materials and composites. Biological processes serve as impressive archetypes of sustainable materials technologies. Because of the potential benefits of biominerals in this regard, its study has gained recognition as an important area of biomimetic materials science (Wakayama et al., 2005).

## Biomineralized carbonates in construction materials

The potential of MICCP technology in restoration of cement mortar cubes, sand consolidation and limestone monument repair, reduction of water and chloride ion permeability in concrete, filling of pores and cracks in concrete, enhanced strength of bricks via urea hydrolysis pathway has been investigated by many researchers (Ramakrishnan, 2007; Sarda et al., 2009; Al Qabany, 2011; Chu et al., 2011; Dhami et al., 2012a; Cheng et al., 2013 and references within).

### Biodeposition in soil and sand materials

**Figure 4:**
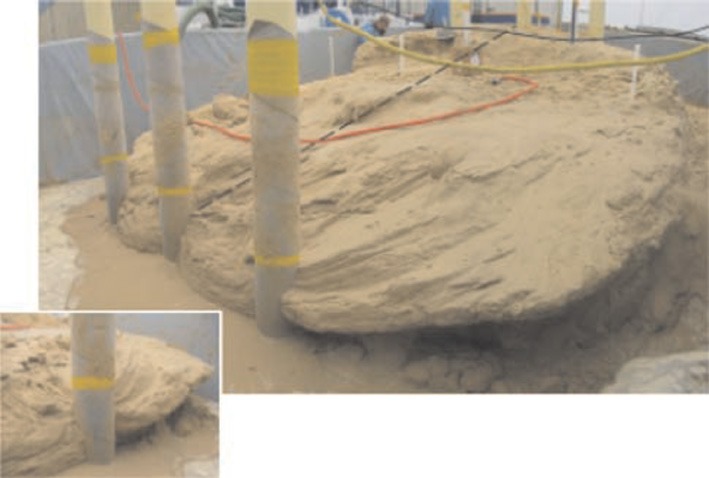
**Image of a cemented sand body from a large scale biogrout experiment. Van Paassen et al. (2010)**.

Mechanical properties of soil are often insufficient and subjected to erosion. Stabilization of soil is highly desirable because of the increasing infrastructure. Conventional chemical grouting techniques are often expensive and require many injection wells for treating large volumes. Recently, the techniques which aims at changing soil properties on demand by stimulating natural (bio-) chemical processes *in situ* has been found and called as biogrouting i.e., *in situ* soil strengthening technique, involving microbial induced carbonate precipitation (Van Meurs et al., 2006; Whiffin et al., 2007; Ivanov and Chu, 2008). In this case, reagents and catalysts are injected and transported to the location where strengthening is required. Many researchers have evaluated the potential of application of bacterially induced carbonate precipitation by ureolytic bacteria by providing urea and calcium sources in various sand plugs (Stocks-Fischer et al., 1999; Bachmeier et al., 2002; Dick et al., 2006; Whiffin et al., 2007; Achal et al., 2009b; Van Paassen, 2009; Van Paassen et al., 2010). Kantzas et al. (1992) reported that sand consolidation by *B. pasteurii* reduced porosity by up to 50% and permeability by up to 90% in the areas where the cementation took place. Improvement in strength of sand columns upon bacterial carbonates was also reported (Ferris and Stehmeier, 1992; De Jong et al., 2006; Whiffin et al., 2007). Recent research initiatives have shown that the calcite crystals form cohesive “bridges” between existing sand grains, increasing the stiffness of sand with limited decrease in permeability (Mitchell and Santamarina, 2005; De Jong et al., 2006; Whiffin et al., 2007; Ivanov and Chu, 2008; Van Paassen et al., 2010; Al Qabany, 2011) (Figure 4). Ureolytic driven MICCP has also been proposed to suppress dust, reduce permeability in granular media, improve soils, stabilize slopes and strengthen liquefiable soils (Gollapudi et al., 1995; Ferris et al., 1996; Whiffin et al., 2007; Bang et al., 2011; Burbank et al., 2011). Harkes et al. (2010) reported the successful potential of MICCP in soil for ground reinforcement. Burbank et al. (2011) subjected the soils on shore Snake river (USA) with ureolytic biomineralization treatments and found around 1% CaCO_3_ in near surface and 1.8–2.4% calcite below 90 cm. Chu et al. (2012) also found considerable reduction in permeability and improvement in shear strength of soil upon application of ureolytic bacterial sp. isolated from tropical beach sand. In the studies of Stabnikov et al. (2011), halotolerant, alkaliphilic *Bacillus* sp. *VS1* successfully sealed a sand-lined model pond. Successive percolation treatments with high concentrations of urea and calcium solutions resulted in a nearly impermeable crust on the surface of the sand, which markedly reduced the seepage rate. Bang et al. (2011) recently showed the potential of MICCP by ureolytic bacteria to suppress dust.

### Biomineralization in cementitious materials

The successful application of bacterial biomineral calcite for improving the compressive strength of mortar and concrete has been reported by several studies (Bang et al., 2001; Ramachandran, 2001; Ghosh et al., 2005; De Muynck et al., 2008a,b; Jonkers et al., 2010; Park et al., 2010; Achal et al., 2011a,c). In all these works, different ureolytic bacteria have been applied (Table 2) and they all have improved the properties of cement concrete specimens to a considerable extent. Ramakrishnan et al. (1998) found increase in resistance of concrete toward alkali, freeze thaw attack, drying shrinkage and reduction in permeability upon application of bacterial cells. De Muynck et al. (2008b) enhanced the permeability characteristics of mortar by *B. sphaericus*. Achal et al. (2011a) treated mortar cubes with *Bacillus* sp. CT-5 and reported nearly six times less absorption of water as compared to untreated specimens. They also studied the effect of *B. pasteurii* on water impermeability in concrete cubes and found the reduction in penetration of water which was more significant on the top side as compared to sides because of better compaction and closing of pores at the top surface (Achal et al., 2011a).

**Table 2:** **Overview of different applications where microbial calcite is used as biocement in cementitious materials**.

**Application**	**Organism**	**Solution**	**Author**
Cement mortar	*Bacillus cereus*	Nutrical	Calcite bioconcept Le Metayer-Levrel et al., 1999
	*Bacillus pasteurii*	SF	Stocks-Fischer et al., 1999; Ramachandran, 2001
Remediation of cracks in concrete	*Bacillus sphaericus*	Growth and biocementation medium	De Belie and De Muynck, 2008
	*Bacillus pasteurii*	SF	Ramachandran, 2001
	*Shewanella*	Sterile	Ghosh et al., 2005
	*Sporosarcina pasteurii*	CSL urea medium, LML urea medium NB urea medium	Mukherjee et al., 2010
Self-healing	*Bacillus pseudifirmus Bacillus cophnii*	Calcium lactate	Jonkers, 2007

### Remediation of cracks in concrete

In case of sealing the cracks in concrete structures, biomineralized CaCO_3_ has proved its efficacy in many reports (Gollapudi et al., 1995; Stocks-Fischer et al., 1999). Bang and Ramakrishnan (2001) used *B. pasteurii* for microbiologically enhanced crack remediation. Similar work was carried out by Ramachandran (2001) who also supported microbiological remediation of cracks in concrete. Bang et al. (2001) encapsulated ureolytic bacterial cells in polyurethanes (PU) and reported positive potential of microbiologically enhanced crack remediation by PU immobilized bacterial cells. De Belie and De Muynck (2008) also reported positive potential of biomineralized calcite in crack repair of concrete by *B. sphaericus*. Qian et al. (2010) reported that compressive strength of biomineralized specimens could be restored to 84%. Recently Achal et al. (2013) reported the positive potential of bacterial calcite in crack remediation by *Bacillus* sp. CT-5.

### Restoration of limestone buildings

MICCP has also been studied for its efficacy in reducing the permeation properties and thereby leading to enhancing the durability of stone specimens by various researchers (Le Metayer-Levrel et al., 1999; Rodriguez-Navarro et al., 2003; Dick et al., 2006). Overview of different methodologies for deposition of microbial calcite layer on stone surfaces is given in Table 3.

**Table 3:** **Overview of different methodologies where microbial calcite has been deposited as a layer on surface of stone**.

**Experimental methods**	**Application procedure**			**Authors**
**Inoculum**	**Bacteria**	**Nutrients**	**Evaluation procedures**	
Culture in exponential phase: 10^7^ to 10^9^ cells/ ml	Spraying	Spraying (5 times)	Water absorption, SEM analysis, surface roughness, colorimetery and plate count	Calcite bioconcept Le Metayer-Levrel et al., 1999
Overnight culture 10^6^ cells cm^−2^	Brushing on water saturated specimens	Wetting every day for 15 days	Water absorption, colorimeteric measurements, stone cohesion	Tiano et al., 1999
2% Inoculums	Immersion in growing bacterial culture (shaking or stationary conditions) for 30 days		Stone cohesion, weight increase, XRD and SEM analysis, porosimetery analysis	Rodriguez-Navarro et al., 2003
1% Inoculums	Immersion in growing bacterial culture (intermediate wetting) for 28 days		Water absorption, SEM analysis	Dick et al., 2006
10^8^ cells ml^−1^	Spraying	In Carbogel	Water absorption and drying due to evaporation	May, 2005
n.d.^*^	n.d.^*^	Immersion in test solution or spraying (*in situ*) tests	Water absorption, colorimeteric measurements, stone cohesion, staining of newly formed calcite with Alizarin Red S and Calcein	Tiano et al., 2006
Overnight culture 10^7^ to 10^9^ cells ml^−1^	Immersion for 1 day	Immersion for 4 days	Weight increase, water absorption, gas permeability, chloride migration, carbonation, freezing and thawing, SEM and XRD analysis	De Muynck et al., 2008a

* n.d., not defined.

It was found that bacterially induced carbonates efficiently reduced the water sorptivity of the treated stone (Le Metayer-Levrel et al., 1999). Rodriguez-Navarro et al. (2003) found that application of *M. xanthus* induces the precipitation of carbonates, phosphates and sulfates in a wide range of solid and liquid media (González-Munoz et al., 1993, 1996; Ben Omar et al., 1995, 1998; Ben Chekroun et al., 2004; Rodriguez-Navarro et al., 2007). Carbonate cementation by this bacterial isolate is successful up to a depth of several hundred micrometers (>500 μm) without any plugging or blocking of the pores. Tiano et al. (1999) investigate the consolidating effect of bacterial biomineral calcite on Pietra di Lecce bioclastic limestone by use of *Micrococcus* sp. and *B. subtilis*. Dick et al. (2006) also reported 50% reduction in water absorption by application of *B. sphaericus* to limestone cubes. The use of carbogel as a delivery system for bacteria for applications in limestone specimens was proposed by Cappitelli et al. (2006a). Zamarreno et al. (2009) investigated the application of calcite crystals precipitated by fresh water bacteria on limestone and found significant reduction in pore sizes of the substrate in treated specimens. De Muynck et al. (2013) reported *B. sphaericus* to be very efficient strain for consolidation of limestone specimens at range of temperatures (10, 20, 28, 37°C). This isolate led to 64% lower weight loss upon sonication and 46% decreased sorptivity in treated limestone specimens compared to the control specimens. De Muynck et al. (2011) recently applied bacterial calcite in two types of stones: microporous and macroporous. They reported that application of bacterial carbonates is more successful in macroporous stone where it occurs to a larger extent and at greater depths than in microporous stone.

### Rescue of buildings of historical interest

Increasing environmental pollution in many areas has been endangering the survival of carbonate stones of historic importance. Architectural and sculptural stones have been seen to undergo deterioration due to several physical, chemical, and biological weathering (Rodriguez-Navarro and Sebastian, 1996; Wakefield and Jones, 1998; Rodriguez-Navarro and Doehne, 1999). A classic example of art under threat was found in the cave of Lascaux in southwest France where devastating infection of *Fusarium* fungus and other molds covered the floor and banks of the main decorated chamber (Rosenbaum, 2006). Other paintings that shared the same fate were found in the Altamira cave in Santillana del Mar, Spain, and the earliest known Christian paintings that adorn Roman catacomb walls. To rescue these and other cultural testimonies, curators teamed up with the idea of recovery and consolidation of works of art by microbial biominerals (González and Saiz-Jiménez, 2005; Cappitelli et al., 2006b). Although microbes were the causative agents for deterioration, they served as the solution also. Several research groups reported that the anaerobic sulphate-reducing bacteria *Desulfovibrio desulfuricans* and *D. vulgaris* efficiently removed the black sulphate crusts that often tarnished buildings (Webster and May, 2006). Other studies reported that the bioformation of oxalic acid generates a protective calcium oxalate patina on stone surfaces (Garcia-Valles et al., 1997). *Shewanella oneidensis* MR1, also inhibited the rate of calcite dissolution under laboratory conditions (Lüttge and Conrad, 2004).

### In low energy building materials

Construction sector is responsible for major input of energy resulting in large share of CO_2_ emissions into the atmosphere (Reddy and Jagadish, 2003). The emission of these greenhouse gases during manufacturing processes of building materials is contributing a lot to global warming. Its time to put emphasis on reducing the emission of these gases into the atmosphere and save energy by minimizing usage of conventional building materials, methods, techniques and working on some other substitutes. For reduction of indirect energy use in building materials, either alternative for bricks, steel and cement have to be found, or vigorous energy conservation measures in these segments of industry have to be initiated. Energy requirements for production and processing of different building materials, CO_2_ emissions and the implications on environment have been studied by many researchers (Oka et al., 1993; Debnath et al., 1995; Suzuki et al., 1995; Table 4).

**Table 4:** **Energy in basic building materials (Reddy and Jagadish, 2003)**.

**Type of material**	**Thermal energy (MJ/ kg)**
Cement	5.85
Lime	5.63
Lime Pozollana	2.33
Steel	42
Aluminium	236.8
Glass	25.8

Reddy and Jagadish (2003) reported soil cement blocks with 6–8% cement content to be most energy efficient building material. These materials have low cost, are easily recyclable and environmental friendly as the soils are mixed with additives like cement, lime etc. As there is no burning involved, this type of stabilized mud blocks help in conserving huge amounts in energy. Dhami et al. (2013b) successfully improved the durability of these low energy building materials by application of ureolytic *Bacillus* sp. This work indicates the potential of this technology for sustainable, cheap and durable buildings.

### Industrial byproduct building materials

As the traditional construction materials (such as concrete, bricks, hollow blocks, solid blocks, pavement blocks and tiles) are all produced from the existing natural resources and damaging the environment due to continuous exploration and depletion of natural resources, many researchers have looked for reusing the wastes in environmentally and economically sustainable ways (Aubert et al., 2006). Different types of wastes along with their recycling and utilization potentials are listed in Table 5.

**Table 5:** **Different types and sources of solid wastes and their recycling and utilization potentials for construction materials (adapted from Pappu et al., 2007)**.

**Type of wastes**	**Source details**	**Recycling and utilization potentials**
Industrial waste (inorganic)	Coal combustion residues, fly ash, steel slag, construction debris	Bricks, blocks, tiles, cement, paint, fine and coarse aggregates, concrete, wood substitute products, ceramic products
Agro waste (organic)	Baggage, rice and wheat straw and husk, saw mill waste, ground nut shell, jute, sisal, cotton stalk	Cement boards, particle boards, insulation boards, wall panels, roof sheets, binder, fibrous building panels, bricks, acid proof cement, coir fiber, reinforced composites, polymer composites
Mining/ mineral wastes	Coal washeries waste, mining waste tailing from iron, copper, zinc, gold industries	Bricks, fine and coarse lightweight aggregates, tiles
Non-hazardous waste	Waste gypsum, lime sludge, lime stone waste, broken glass and ceramics	Blocks, bricks, cement clinker, hydraulic binder, fibrous gypsum boards, gypsum plaster, super sulfated cement
Hazardous waste	Contaminated blasting materials, galvanizing waste, metallurgical residues, sludge from waste water and waste water treatment plants	Boards, bricks, cement, ceramics, tiles

Fly ash (FA) generated during the combustion of coal for energy production is one of the industrial byproduct that is recognized as an environmental pollutant. Rice husk ash (RHA) obtained from burning of rice husk is another major agricultural byproduct and available in all parts of the world except Antarctica (FAO, 2002). Both these materials can be used successfully in construction materials such as bricks and blocks without any degradation in the quality of products (Nasly and Yassin, 2009) but problems associated with ash bricks are low strength, higher water adsorption, low resistance to abrasion, low fire resistance and high porosity (Kumar and Palit, 1994). Dhami et al. (2012b) studied the application of bacterial calcite on these ash bricks (FA and RHA) and found it to be very effective in reducing permeability and decreasing water absorption leading to enhanced durability of ash bricks.

## Challenges and issues

The field of biomineralization includes a multidisciplinary research involving experts from various fields. Though its potential has been suggested in a variety of sectors but several efforts need to be made to address key research and development questions necessary for commercial scale applications.

One of the disadvantages of microbial cementation method in comparison with chemical methods is that the microbial process is usually slower. This method is more complex than the chemical one as the microbial activity depends on many environmental factors including temperature, pH, concentrations of donors and acceptors of electrons, concentrations and diffusion rates of nutrients and metabolites etc. Due to this complexity, its usage at large-scale has not been so encouraging. Other gaps with usage of calcinogenic microbes include inconvenient application procedures, development of other unwanted microbes as repeated application of nutrients conceivably permit their development, need for detailed microbial ecology studies in order to ascertain the effects of the introduction of new bacteria etc.

The economic limitation of use of laboratory grade nutrient sources in field applications also restricts the use of this technology in several cases. Successful commercialization of the technique requires economical alternatives of the medium ingredients that cost as high as 60% of the total operating costs (Kristiansen, 2001). Use of inexpensive materials as corn steep liquor (CSL) or lactose mother liquor (LML) may help to lower the cost of treatment (Achal et al., 2009a,b, 2010; Mitchell et al., 2010). Production of large volumes of reactants and cultures also makes this technology a bit inconvenient as compared to traditional treatments.

Production of ammonia during hydrolysis of urea poses environmental concern (toxic effects on human health, vegetation, atmospheric nitrogen deposition, leading to the eutrophication and acidification of sensitive ecosystems) as well as leads to discoloration of stone (Sutton et al., 2008; Tobler et al., 2011). Additional research is necessary to overcome this problem and encourage the use of MICCP for production of biominerals.

The survival of bacteria within the building material also influences the extent of calcification. Detailed microbial ecology studies are imperative in order to ascertain the effects of the introduction of new bacteria into the natural microbial communities, the development of the communities at short, mid and long-term, and the eventual secondary colonization of heterotrophic microorganisms using bacterial organic matter and dead cells, such as actinomycetes, fungi, etc.

The above mentioned concerns limit the use of MICCP for practical applications in various fields in comparison to the traditional methods.

## Concluding remarks and suggestions for future work

Microbially induced biominerals by microbes are being used extensively these days in various fields ranging from removal of heavy metals and radionucleotides to remediation of building materials. The potential of these biominerals has brought a new revolution in various engineering applications but still there has been much to explore in order to bring this environmentally safe, cost effective and convenient technology from lab to field scales. More exploratory works at large scale should be undertaken to determine the efficacy of *in situ* biomineralization for sequestration of metals, PCBs, CO_2_ sequestration as well as consolidations of buildings. Comparative studies should be done to check the feasibility of this method with that of the chemical methods which should include environmental impacts as well as cost. The optimal balance of substrates used for various applications should be looked upon to increase the economic feasibility and reduce the production of unwanted byproducts. Long term efficacy of these biominerals should be investigated. Efforts should also be made to improve current mathematical models for determining MICCP at macro-scales. As the successful implementation of MICCP-based technologies require experts of varying disciplines, researchers from all around the globe should work together to make this multi-disciplinary research move toward commercial scale applications at a higher pace.

### Conflict of interest statement

The authors declare that the research was conducted in the absence of any commercial or financial relationships that could be construed as a potential conflict of interest.
